# Antipyrine as a Hæmostatic

**Published:** 1888-03

**Authors:** W. M. Powell

**Affiliations:** Albany, Texas


### ANTIPYRINE AS A HÆMOSTATIC.

*By W. M. Powell, M. D., Albany, Texas.*

For Daniel's Texas Medical Journal.

This drug, I believe, was first introduced as anti-pyretic, but it seems to be a more valuable agent in other respects, at least, it has given me better results. In fevers I regard it unreliable, because, even in moderate doses, it depresses the patient to such a degree of prostration that stimulants must necessarily be promptly given. Dr. O. L. Williams, of Chapel Hill, Texas, in a paper read before the State Medical Association, calls attention to its value in sick headache, and I can also testify to its good effects in that troublesome disease, having used it (uncombined with other drugs) in 10–15 gr. doses before I read Dr. Williams' interesting article.

Now, as a local hæmostatic I can only briefly refer to two cases that came under my care recently. Mrs. S. is the victim of an "old sore leg" of some three years standing, the sore situated on anterior surface, lower third of tibia. Some time ago, while out in the dark drawing water she accidentally struck the sore leg against the spout of a stove kettle which caused a frightful hemorrhage, filling her shoe full of blood in a very short time. I was sent for in haste, and when I arrived found her neariy exhausted. I applied a four per cent solution of antipyrine and had the satisfaction of seeing the bleeding quickly checked. I then applied a light compress and bandage and elevated the extremeties on pillows in bed. No more hemorrhage has since occurred.

A few days later I operated on a boy in his seventh year, for phimosis, removing nearly one inch of the prepuce; the hemorrhage, of course, was profuse. Before removing the clamp forceps I applied a four per cent solution of antipyrine, also immediately after removing them. All bleeding was promptly arrested and the mucous membrane and foreskin were neatly brought together with numerous stitches without the least annoyance from further hemorrhage. A simple water dressing completed the operation, and on redressing, the following day, I do not think I ever saw a cleaner, nicer wound.

Dr. Cosati, in Independencia Medica, concludes as follows:

1. "Antipyrine is a powerful hæmostatic."

2. "It is superior to perchloride of iron, because it leaves the wound perfectly clean."

3. "It is even superior to ergotine, because it has no toxic effects if the doses are not too enormous."

5. "In most cases it is preferable on account of its double antipyretic and antiseptic action."

6. "The hæmostatic action takes place in a very short time."

My little experience with the drug thus used induces me to fully concur with this writer, and I would be glad to hear from others who may have used it in the same way, as it is comparatively a new drug, and I am not familiar with its origin and general use. As above stated, it has not given satisfaction in fevers in my hands, and I purpose at some future time to write up my experience with it in typhoid and other varieties of fevers.

